# The Disentanglement of the Body–Consciousness–World Intertwining: A Phenomenology of Severe Mental Illness

**DOI:** 10.1111/nin.70134

**Published:** 2026-07-16

**Authors:** Rui Gomes, Patrícia Silva‐Pereira

**Affiliations:** ^1^ Nursing Research Innovation and Development Centre of Lisbon (CIDNUR), School of Nursing Universidade de Lisboa Lisbon Portugal

**Keywords:** embodiment, interpersonal relations, lived experience, phenomenology, psychiatric nursing, severe mental illness

## Abstract

Severe mental illness is frequently approached through nosological criteria focused on symptom severity, neglecting the complexity of lived experience. Phenomenology understands psychopathology not as an isolated biological dysfunction, but as a radical alteration in the structure of being‐in‐the‐world. Grounded in Merleau‐Ponty's ontology and Max van Manen's phenomenology of practice, this article presents partial findings from a broader study, aiming to unveil the lived experience of the body–consciousness–world intertwining in persons with severe mental illness. To this end, phenomenological conversational interviews were conducted with 12 clinically stabilized adults attending a community institution, prioritizing the depth and phenomenological richness of the accounts. The experiential material was analyzed through epoché, eidetic reduction, and vocative writing. Findings unveil that the illness emerges as a disentanglement, materialized in the essential theme: “a disentanglement in multiple forms of body–consciousness–world metamorphosis.” The experience manifests through a violent rupture of the intentional arc: the body loses its transparency and the world its familiarity, yielding lived experiences of withdrawal, corporeal doubt, blackout of consciousness, and a fracturing of intercorporeality. This highlights the urgency for a humanistic nursing that transcends symptom management and embraces the ethics of welcoming, establishing itself as an ontological mediation that validates the person's lived experience and promotes the re‐intertwining of new possibilities of being‐in‐the‐world.

## 1 Introduction

Severe mental illness is usually framed by a cluster of signs and symptoms categorized into nosological entities. Clinically defined by criteria such as the DSM‐5 or ICD‐11, severe mental illness encompasses conditions like schizophrenia, bipolar disorder, and major depression, characterized by symptom severity, chronicity, and a profound impact on social and occupational functioning (Oliveira Martins et al. 2022; Tse and Haslam 2023).

This approach, by focusing on the *disease*—that is, the pathology as a measurable entity separate from the person—tends to reduce individuals to an objective body (*Körper*), neglecting the lived body (*Leib*) and the uniqueness of subjective experience (J. Aho and Aho 2008; Frohn and Martiny 2023; Husserl 2013). By treating pathology as a deviation from a statistical or physiological norm, there is a risk of promoting the objectification of the body (SmithBattle and L'Ecuyer 2025), transforming it into a mere object of analysis and pharmacological modification (K. Aho 2021; Escribano 2024). Such a perspective highlights the body as a mechanism to be repaired, obscuring the personal dimension of care.

Thus, an exclusive reliance on categorical criteria proves insufficient to capture the subjective nature of these disorders, frequently resulting in a gap between codified clinical reality and first‐person phenomenological experience (Peraire et al. 2023; Van Heugten‐Van Der Kloet and Van Heugten 2015), where the *illness*—the subjective experience of not feeling well—often remains in the shadows (Carel 2016; Cosgrove and Suppes 2013). This gap invites mental health nursing to establish itself as a relational anchor, a grounding presence that transcends the technical surveillance of symptoms to embrace lived experience in its radical vulnerability. Understanding how the being inhabits a fractured world grounds a practice pathically attuned to the person's experience. In this context, phenomenology emerges as a fundamental philosophical and methodological pathway to return to the things themselves, as proposed by Husserl and deepened by Maurice Merleau‐Ponty (Merleau‐Ponty 2020)—a necessary complement that prioritizes the subjective reality of experience (Spencer et al. 2025).

Grounded in the Merleau‐Pontyan notion that human existence is a body–consciousness–world intertwining (Verano Gamboa 2025; Merleau‐Ponty 2019), this article aims to unveil the lived experience of persons with severe mental illness, focusing on understanding the alterations in this primordial intertwining. It presents partial findings from a broader phenomenological‐hermeneutic study. We focus specifically on exploring how the disruption of the primordial bond between body, consciousness, and world inaugurates an itinerary of suffering and strangeness.

By adopting a perspective that values the chiasmic dimension of the flesh in its porosity and reversibility (Verano Gamboa 2025; Merleau‐Ponty 2019), we seek to contribute to a more humanistic, ethical, and responsive mental health and psychiatric nursing, capable of welcoming the face of the Other in their radical vulnerability (Levinas 2014; Watson 2002). This inquiry responds to the urgent need to transcend symptom management to understand the “how” of lived experience, legitimizing this experience not as an account of deficits, but as the expression of an existence striving to resignify itself in a world that has grown strange. This approach does not imply a rejection of psychiatric diagnoses or neurochemistry. On the contrary, the expansion of the clinical gaze requires the integration of biology into the living structure of human existence, recognizing that care is not exhausted in the correction of a physiological deviation, but in facilitating a new possibility of being‐in‐the‐world (K. Aho 2021). Within this framework, physiological and experiential processes are understood in a mereological (part‐whole) relationship: while medication may address the local, physiological layer, phenomenology focuses on the global, existential layer of the person as a whole (De Haan 2020).

## 2 Theoretical and Philosophical Framework

Understanding the lived experience of severe mental illness demands a rupture with the Cartesian dualism that has historically cleaved the human condition into *res cogitans* (mind) and *res extensa* (body). Moving beyond the biomedical view that reduces the person to a biological organism, Maurice Merleau‐Ponty, in his *Phenomenology of Perception* (2020), presents us with a return to the things themselves. Phenomenology does not reject science; rather, it seeks to unveil the lifeworld (*Lebenswelt*) that precedes it, illuminating the birth of meaning within pre‐reflective experience. The theoretical and philosophical concepts mobilized throughout this study, particularly those of Merleau‐Ponty and other classical authors, were interpreted and translated into English by the authors from the editions listed in the references, ensuring the preservation of their originary phenomenological nuances.

### 2.1 The Primacy of Perception and Embodied Consciousness

The cornerstone of this phenomenology lies in the ontological rehabilitation of the body. Countering both intellectualism and empiricism, Merleau‐Ponty (2020) postulates that *I am my body*, understanding it not as an object to be possessed, but as the very condition of possibility for existence. Originally, consciousness is not an “I think that,” but an “I can,” with the body serving as the vehicle of being‐in‐the‐world, the zero‐point of all orientations, and the origin of all significations.

This notion also mobilizes the fundamental phenomenological distinction, inherited from Husserl, between the objective body (*Körper*) and the lived body (*Leib*) (J. Aho and Aho 2008; Carel 2016). While traditional medicine frequently focuses on the *Körper* as an anatomical, neurochemical entity measurable in the third person, the experience of falling ill unfolds within the *Leib*. The lived body is the embodied, volitional, and sentient consciousness through which we inhabit the world. As Merleau‐Ponty (2020) metaphorically states, the lived body is to the world as the heart is to the organism: it keeps the visible spectacle continuously alive, animating and nourishing it from within.

In this study, the triad body–consciousness–world is understood as a chiasmic structure that constitutes the very foundation of being‐in‐the‐world (Merleau‐Ponty 2019). By consciousness, we do not refer to a disembodied mental faculty or a purely cerebral function, but to an embodied ipseity—the basic, pre‐reflective sense of self that is inseparable from the flesh (Fuchs 2010; Zahavi 2014). Consequently, the radical disruption of this intertwining precipitates a profound existential crisis, where the fragmentation of self‐coherence and the loss of a meaningful world emerge as two sides of the same phenomenon (De Haan 2020; Stanghellini 2016).

### 2.2 The Intentional Arc and Transparency in Health

In a healthy existence, the relationship between the body and the world is sustained by what Merleau‐Ponty designates as the intentional arc. Intentionality, in this context, is understood as the basic structure of consciousness always being directed toward the world through the body's powers. Crucially, consciousness is not an isolated container, but a carnal commitment—a dynamic structure that projects the person toward their past, their future, and their human milieu, unifying the senses, intelligence, sensibility, and motility into a coherent whole (Merleau‐Ponty 2020).

It is this arc that guarantees the body's transparency: in health, the body operates with a transparency that allows for a pre‐reflective absorption in the world, functioning as a silent ally (Sartre 2021) or an absence (Leder 1990). As Gadamer (2009) notes, health resides in its capacity to remain hidden, in a self‐forgetfulness that allows for the fluidity of action. The healthy body relies on a dynamic body schema and a habituated know‐how that allows us to interact with the world without the need for constant reflection (Carel 2016).

However, in severe mental illness, this arc slackens or distends, leading to a disentanglement where the primordial “I can” is replaced by a conspicuous opacity. The unity of experience fragments, and the body emerges as an obstacle or an alienating presence, stalling the flow of existence and transforming the world into an inhospitable space (De Haan 2020; Fuchs 2005; Merleau‐Ponty 2020).

### 2.3 The Ontology of the Flesh (*La Chair*) and the Chiasm

To deepen the nature of this intertwining, Merleau‐Ponty's late work, *The Visible and the Invisible*, offers his ontology of the flesh (*chair*). The flesh is neither matter, nor spirit, nor substance; it is a primordial ontological element, much like water or fire (Verano Gamboa 2025; Merleau‐Ponty 2019)—the common elemental tissue that constitutes both the seeing body and the visible world, allowing them to communicate.

The relationship between the body and the world is thus configured as a chiasm (*chiasme*): an intertwining or crisscrossing where boundaries become porous. Merleau‐Ponty (2019) describes this phenomenon through reversibility: we are seeing beings who are visible, touching beings who are tangible, where my body is made of the same flesh as the world. We are not merely in front of the world, we are *of* the world; the body uses its own being as a means to participate in the being of things, “making itself world and making the things flesh” (Merleau‐Ponty 2019).

This ontology of intertwining grounds the central thesis of this article: in severe mental illness, a disentanglement occurs within this chiasmic tissue. Reversibility becomes blocked or distorted, leading to the loss of vital contact with reality described by Minkowski (2004) or to the radical corporeal doubt identified by Carel (2016), where the tacit certainty of inhabiting a reliable world collapses.

### 2.4 Intercorporeality and the Ethical Dimension

The intertwining inevitably extends to the social sphere, into a structure of intercorporeality (Merleau‐Ponty 2019). Recognizing the Other is not an intellectual act, but an immediate bodily resonance, an interaffectivity sustained by a common flesh (K. Aho 2021; Fuchs 2017). Our bodily fields are sensibly intertwined, allowing us to intuitively understand the gestures and emotions of others.

In psychopathology, this “musical memory” of interaction falls out of tune (Fuchs 2017). The gaze of the Other, which should confirm existence, can become objectifying or threatening. This vulnerability summons the ethics of Levinas (2014): the Face of the Other, in its nakedness and exposure, is not an object of knowledge, but an ethical imperative that commands responsibility. Phenomenological nursing is founded upon the recognition of this shared vulnerability and the responsibility to welcome the radical alterity of the one whose world has grown strange.

### 2.5 Psychopathology as a Stalled Existence

Mobilizing this framework allows us to understand severe mental illness as structural modifications of being‐in‐the‐world, oscillating between poles of corporeal opacity described by Thomas Fuchs (2005, 2010). On one hand, corporealization, often associated with depression, occurs when the body loses its lightness and becomes a heavy, “hyper‐embodied” object that resists intentionality and stalls lived time (Bizzari 2023). Conversely, the pole of disembodiment, frequently linked to schizophrenia, is characterized by the person losing their tacit immersion in the body, becoming a disembodied spectator of their own biological machinery. In this state, an erosion of ipseity and a fragmentation of operative intentionality occur (Feyaerts and Sass 2024; Kyzar and Denfield 2023; Spencer et al. 2025), inaugurating a metamorphosis where intercorporeality fractures (Stanghellini 2009). Within this framework, severe mental illness presents itself as a metamorphosis of the intertwining—an existential crisis where the continuity of meaning and trust in the world are ruptured (Brijan et al. 2025; Fusar‐Poli et al. 2022, 2023).

## 3 Methodological Pathway

The method in this inquiry is inextricably linked to the fundamental question posed by Merleau‐Ponty (2020) in the preface to the *Phenomenology of Perception* regarding the true nature of phenomenology. For the author, the answer is far from being found in theoretical instances; rather, it resides in subjectivity itself (the perceived world) and in the continuous effort to return to the things themselves. This study assumes that the function of inquiry is to replace facticity within the field of private experience where it germinates, illuminating its birth.

To operationalize this return to the pre‐reflective world of the lived experience of persons with severe mental illness, Max van Manen's Phenomenology of Practice (2014, 2023) was mobilized. This approach is distinguished by its agogic character (from the Greek *agogos*, guide or pathway), which implies that the method does not crystallize into a rigid set of rules, but rather illuminates a singular possible path. It is an investigative pathway that aims to establish formative relations between being and acting, privileging a pathic understanding of the world—a knowledge that is more relational, situational, corporeal, and temporal, transcending merely technical or gnostic rationality (Van Manen 2007, 2014).

### 3.1 The Phenomenological Attitude: *Epoché* and Reduction

Methodological rigor was grounded in the dual and complementary movement of *epoché* and reduction. *Epoché* constitutes the negative moment of suspending the beliefs, theories, and natural attitudes that obscure the phenomenon, while reduction represents the positive movement of leading the gaze back (*reducere*) to the original mode of appearance of the experience (Van Manen 2014). Following the Van Manenian methodological architecture, this movement unfolds into specific dimensions that guided the researcher's posture, integrating a heuristic reduction (wonder), which involves the awakening of a profound sense of wonder toward the phenomenon, seeking to see the extraordinary in the ordinary and allowing the lived experience of the illness to speak in its radical novelty. This is complemented by a hermeneutic reduction (openness), requiring the critical effort of self‐consciousness to suspend prior theoretical and psychiatric assumptions (the traditional “clinical gaze”), ensuring genuine openness to what gives itself. Furthermore, an experiential reduction (concreteness) was applied to maintain the orientation toward the concreteness of lived experience (time, space, and body) preventing explanatory abstractions from overriding the immediacy of the phenomenon during the gathering of experiential material. Concurrently, eidetic reduction directed the search for the invariants or *eidos* of the phenomenon, distinguishing between essential and incidental themes through free imaginative variation, questioning whether the phenomenon would lose its fundamental meaning if a certain attribute were removed.

### 3.2 Context and Participants

The gathering of lived experience descriptions took place in collaboration with a community psychosocial rehabilitation institution. Access to participants involved prior collaborative work with the institution's technical teams to identify individuals whose experiences were rich and relevant to the phenomenon under study. This selection was intentional, aiming to ensure experiential richness and a plurality of life experiences. In a subsequent phase, the researcher established direct contact with the participants to gauge each individual's motivation. There was no prior therapeutic relationship; the researcher established initial contact specifically for the study's purposes, following the institution's mediation.

Twelve clinically stabilized adults (aged 28–61 years, with illness duration ranging from 7 to 45 years), diagnosed with severe mental illness (schizophrenia, bipolar disorder, and major depression) and capable of giving free and informed consent, participated in this inquiry. All twelve invited individuals agreed to participate, with no refusals. The intentional invitation safeguarded the phenomenological richness of the study. In congruence with the phenomenological method—which seeks to unveil the experience rather than characterize the participants—rigor was not based on inferential or exhaustive demographic analyses, but on eidetic rigor through reduction and imaginative variation (Van Manen 2014). For the presentation of illustrative episodes in the findings, pseudonyms were assigned to the participants.

Interviews took place in locations chosen by the participants (e.g., gardens, well‐lit rooms) to ensure comfort and privacy. Efforts were made to foster a relational atmosphere of sharing, closeness, safety, and trust prior to the interview. The interviews were conducted individually, one‐on‐one, with only the researcher and the participant present. Each participant was interviewed only once, without follow‐up interviews, given the focus on the experiential density of each encounter.

### 3.3 The Conversational Interview

The privileged empirical method for gathering experiential accounts was the phenomenological conversational interview, focused on eliciting descriptions of concrete lived experiences. The interviews varied in length from 43 to 92 min; they were audio‐recorded and transcribed verbatim, constituting the raw material for analysis. The original transcripts were analyzed in their native language to preserve phenomenological nuances, and the selected illustrative episodes were translated into English by the authors, ensuring the preservation of the original pathic and embodied meaning. Field notes focusing on non‐verbal language and the atmosphere of the encounter were recorded immediately after each interview. These field notes complemented the thematic analysis, informing the understanding of the embodied dimension of the experience. In this context, the interviews were conducted between April and August 2022 by the researcher (male), who is trained in mental health and psychiatric nursing, a nursing doctoral candidate, and holds advanced competency in psychotherapy from the national regulatory authority.

This professional background, although facilitating active listening and the emotional containment of the participants, held the potential to generate a perceived authority bias (“the nurse will judge/medicate me”), which was carefully mitigated. The *epoché* operated precisely as a counterweight. Specifically, it involved the suspension of clinical knowledge, where diagnostic and therapeutic assumptions were recorded in pre‐interview reflective notes and suspended during the encounter, prioritizing the lived experience over nosological categories. The researcher engaged in a reflexive *epoché*, documenting prior clinical and personal assumptions to suspend judgments, whether diagnostic or otherwise. Concurrently, this posture demanded the containment of the therapeutic impulse, wherein the desire to intervene was identified and contained by the strict methodological intention of non‐interventional understanding, explicitly communicating to the participants: “This conversation is solely to listen to your experience, not to treat or advise.”

This tension enriched the researcher's pathic sensibility, capturing silences and bodily hesitations that precede the word in lived experience. Thus, the phenomenological dialogue transcended instrumental logic, safeguarding the necessary openness for the phenomenon to unveil itself in its alterity and original integrity.

Consequently, the objective was not to gather opinions or interpretations, but to obtain descriptive accounts of pre‐reflective lived experiences (Van Manen 2014) through open‐ended prompts such as “Tell me about…,” “How did you feel in that moment?,” or “Give me an example.” A return to the lived moment was encouraged, seeking concrete details about spatiality, temporality, and corporeality, while avoiding abstract generalizations.

The interview transcripts were not returned to the participants, given the phenomenological focus on the lived experience and reflective analysis rather than on the participant as a person, in accordance with Van Manen (2014), who likewise emphasizes the sense of plausibility of the lived experience over its strict facticity.

### 3.4 Reflective Analysis and Vocative Writing

In the phenomenology of practice, analysis is not a mechanical process separate from writing; writing is the very method of inquiry and revelation (Van Manen 2014). Thus, a crafted, artisanal process of writing and rewriting unfolded, in which the way the interview contents were engaged followed a rigorous trajectory. This process involved constructing the illustrative episode (anecdote), where raw material from the interviews was edited to create illustrative episodes or phenomenological anecdotes. These short, bare descriptions aim to capture the incident precisely, culminating in a *punctum*—an element that touches and summons the reader to reflection, mediating the intuitive understanding of the experience's singularity (Van Manen 2014). Alongside this, thematic analysis was performed, applying three reading approaches to recover meaning structures: the holistic approach (capturing the fundamental meaning of the text in a single phrase), the selective or highlighting approach (identifying essential phrases that stand out), and the detailed or line‐by‐line approach (interrogating the intrinsic meaning of each sentence) (Van Manen 2014, 2016).

The trajectory also integrated eidetic reduction and imaginative variation, where emerging themes were subjected to imaginative variation to distinguish the essential from the incidental, seeking the invariant that makes the phenomenon what it is (Van Manen 2014). Furthermore, vocative writing was employed in the elaboration of the phenomenological text, using the philological method and utilizing evocative, invocative, and provocative language. The aim was to produce a text with pathic resonance, capable of *speaking the world* rather than merely speaking about it, allowing the experience of the intertwining in severe mental illness to become intelligible not only cognitively, but sensorially and affectively (Merleau‐Ponty 2020; Van Manen 2014).

The analytical process was not linear, but rather a cyclical and continuous movement of phenomenological writing and rewriting, as advocated by Van Manen. Initially, the selective or highlighting approach was used to identify phrases or expressions that captured the essence of the lived experience of the intertwining phenomenon. These units of meaning were clustered into thematic nodes and subsequently transformed into phenomenological anecdotes, which served as the basis for the eidetic reduction. Successive rewriting did not aim merely at stylistic refinement; it functioned as a method for discovering meaning and for constant reflection, allowing the pathic significance of embodiment and the lifeworld to emerge beyond the factual description of symptoms. The analysis was conducted manually and artisanally, without the use of qualitative data analysis software, prioritizing the researcher's deep immersion in the narrative texts and the pathic sensibility required to apprehend the phenomenological themes.

### 3.5 Rigor and Ethical Considerations

The analysis followed Van Manen's holistic, selective, and detailed reading approaches, where emerging themes and illustrative episodes were periodically reviewed and discussed between the researcher and the two members of the supervisory team—who hold expertise in philosophy/phenomenology and mental health and psychiatric nursing—to validate interpretative consistency. These were further submitted to scrutiny within a Collaborative Analysis Group comprising three experts in nursing, phenomenology, and mental health, who provided new insights, validation, and resolution for any potential moments of divergence, impasse, or doubt. Thus, the analysis benefited from continuous critical review and intersubjective validation, ensuring that the findings reflect a collective reflexivity rather than a subjective construction. Furthermore, the robustness of the analysis was supported through the use of insight cultivators—philosophical, literary, and theoretical sources that operated as interpretative catalysts, opening new possibilities for understanding without imposing theory upon the lived experience (Van Manen 2014).

Regarding the ethical dimension, the study was approved by the competent Ethics Committee of the authors' affiliated institution (Process 3624/2021/CE), and free and informed consent was obtained from all participants. This process guaranteed anonymity, confidentiality, and respect for each individual's vulnerability, ensuring that the inquiry served as a safe space for sharing and recognizing lived experience in the first person. The preparation of this article also considered the Consolidated Criteria for Reporting Qualitative Research (COREQ‐32) as a framework to ensure transparency. However, being a phenomenological‐hermeneutic study mobilizing Max van Manen's phenomenology of practice, methodological rigor was not limited to procedural adherence to a checklist; it was established primarily through Van Manen's (2014) criteria of orientation, strength, richness, and depth. Accordingly, necessary adaptations were made to accommodate the specificity of the inquiry, privileging interpretative density over technical standardization.

## 4 Findings: A Body–Consciousness–World Disentanglement

The phenomenological analysis of the interviews unveiled that the lived experience of severe mental illness presents itself as a structural transformation of being‐in‐the‐world, whose essence resides in the rupture of the body–consciousness–world intertwining. Without this rupture, the phenomenon of the lived illness does not manifest, leaving perhaps only the biological condition (*disease*). This section presents the essential theme “a disentanglement in multiple forms of body–consciousness–world metamorphosis” and the five sub‐themes that unveil the multiple faces of this metamorphosis.

The illness presents itself as an overwhelming event that violently disrupts the pre‐reflective unity of existence. The concept of disentanglement refers to the act of undoing or loosening what was united—the vital knot between body, consciousness, and world. Meanwhile, “metamorphosis,” from the Greek *metamorphōsis*, designates a radical change in form or structure. As Byung‐Chul Han observes, “anguish is a threshold feeling… The threshold as a place of metamorphosis hurts” (Han 2022, 61). The disentanglement is situated precisely at this threshold of pain, where a completely different state of being begins.

Just as in Franz Kafka's literary work *The Metamorphosis* (2023), where the protagonist Gregor Samsa awakens transformed into a monstrous insect, the participants in this study describe an alteration where the body ceases to be “standard” and spatial and temporal relations undergo drastic mutations. The body becomes strange, alien to the domain of the will, inaugurating an existence where identity dissolves, and familiarity collapses.

From the essential theme that manifested, five sub‐themes emerged, unveiling the multiple faces of this metamorphosis. These themes describe a progressive journey of suffering, beginning with the loss of totality in *a fully stretched heart* and moving toward the inertia of withdrawal in *I couldn't get out of bed*. This trajectory is further deepened by the ontological instability of *very strange things* (corporeal doubt) and the social alienation found in the *everyone is looking at me* experience (rupture of intercorporeality), ultimately culminating in the profound disconnection of *I walked around completely numb* (blackout of consciousness). In the following sections, we will explore each of these sub‐themes through their illustrative episodes and radical reflection, mapping an itinerary of existence where the familiar world gradually loses its meaning and the individual struggles to resignify themselves amidst radical vulnerability.

### 4.1 The Loss of Totality: A Fully Stretched Heart

The metamorphosis frequently begins in the intimacy of the flesh. In Roberto's experience, the disentanglement manifests through an altered corporeal perception that marks the beginning of the rupture with the habitual world:

It all started with an interrupted sleep disturbance. I started hearing voices, and that bothered me a lot. I felt like a dead person, with no productivity at work, with a lot of confusion in my head… My heartbeat speeding up thump thump thump. Sometimes feeling short of breath, sometimes having leg dislocations while walking, strange things! The heart was fully stretched.

(Roberto)

Roberto's body ceases to be the *silence of the organs* characteristic of health. The *fully stretched heart*, the palpitations, and the *strange things* signal the transition from the transparent body (*Leib*) to the opaque body. The presence of voices invades the intimate spatiality of consciousness, generating confusion and a sensation of death in life. The body is lived as an opponent, something stretched to the limit, illustrating the onset of an unbearable tension where psychophysical unity fragments.

### 4.2 Withdrawal and the Virtual World: *I Couldn't Get Out of Bed*

In João, the metamorphosis of the intertwining takes the form of inertia and refuge. The world progressively withdraws, and the bed becomes the gravitational center of a stalled existence:

I was already spending whole days in bed… Very quickly, hygiene at home deteriorated. Very quickly I stopped cooking… the only thing I could order was pizzas, I ate pizza every day. I started taking refuge in video games… I created an “ideal persona”. On the computer, to those people, I was a very intelligent medical student. But I was in my pajamas at home, and I hadn't been out for 3 or 4 months. I was in bed with a level of disorganization, with pizza boxes up to the ceiling. Being in bed is comfortable and it is extremely depressing. I couldn't get out of bed. I started getting fatter.

(João)

Here, the disentanglement occurs within operative intentionality. The everyday know‐how regarding hygiene or cooking degrades, and the physical body—now fat and surrounded by disorganization—becomes a burden. To escape this corporeal impotence, João projects himself into an ideal digital persona. A schism occurs between the inert lived body (in pajamas, in bed) and a consciousness attempting to inhabit a virtual world where it is still possible to be intelligent and capable. The bed, paradoxically comfortable and depressing, becomes the site of a cycle of sorrow where future time is suspended and the present slips away.

### 4.3 Corporeal Doubt and Permeability: *Very Strange Things*

For Mafalda, the metamorphosis is sensory and dissolves the boundaries between self and world, instilling a radical doubt regarding the reality of her own perception:

I saw dirt in various places… there were various smells, I smelled everything normal and everything abnormal. There were smells that… it was impossible for those smells to be there. Like rotten… rotten fruit. Like sweat. Very strange things like that… I think the smell of sweat was from my own body. But I showered! Once I was taking a shower and I heard them say: “Look, wash with this shampoo”. I heard my neighbors saying that. I felt everyone touching me. I would walk past someone and feel like I was getting slapped.

(Mafalda)

Mafalda's experience unveils a collapse of tacit trust in the body. The pre‐reflective certainty of inhabiting a reliable world crumbles in the face of impossible smells and invisible touches, felt as real. The boundary between the private (the shower) and the public (the neighbors' voices) becomes permeable. The lived body ceases to be a secure boundary to become an invaded territory, where the distinction between what is mine (the sweat) and what belongs to the world becomes diffuse, plunging Mafalda into a profound ontological disquiet that leads her to seek answers everywhere (“I even went to a traditional healer”), such is the strangeness of the experience.

### 4.4 The Rupture of Intercorporeality: *Everyone Is Looking at Me*

In Maria's exemplary case, the disentanglement manifests in the relationship with the Other. Intercorporeality, habitually a space of mutual recognition, metamorphoses into a space of threat and persecution:

I was on the bus. The air was heavy. There was a lot of pressure and my heart was speeding up with palpitations. “Everyone is looking at me!”. People all looked at me and started speaking ill of me. I pulled away from everyone! I shrank into a corner, like a frightened animal. “That one is sick, she's sick in the head!”. I feel like everyone is looking at me. And they start laughing.

(Maria)

The social world becomes hostile. The reversibility of the gaze inverts: it is perceived by consciousness as a personal attack. The *frightened animal* shrunk in the corner of the bus illustrates the collapse of basic trust in the social fabric. The presence of the Other ceases to be co‐existence to become judgment, forcing Maria into flight and isolation.

At the movie theater, the experience repeats itself:

When I went to the movies, people would start laughing. But the movie was a comedy. Except I thought people were laughing at me. I had to leave. I know people are speaking ill of me.

(Maria)

Here, the laughter of others reinforces an attacked consciousness. The bond that unites the person to humanity ruptures, and the world becomes a place of persecution.

### 4.5 The Blackout of Consciousness: *I Walked Around Completely Numb*

The most radical form of disentanglement emerges in the illustrative episodes of Mário and Bianca: the blackout or total disconnection. Here, consciousness seems to be torn away, and the body wanders in a seemingly meaningless automatism.

Mário describes the experience of living on another planet:

I walked around completely numb. I had no awareness of anything, I don't even know how I crossed the streets. I saw people at home, but no one was there. I talked to myself… things that made no sense at all. For four months I slept on the street. I had my house keys in my pocket, I had my transit pass, but I didn't know how to go home. I felt no cold at all. I was blocked, completely trapped in a world I can't explain. It's like being enclosed in a sphere… I was in a total blackout: no memory, no life, no consciousness. A world apart. Another planet, planet madness.

(Mário)

Bianca shares an experience of loss of self:

That period of 3, 4 days… I have a blackout in my mind. I lost my sense of reality and completely lost my sense of time. I was walking barefoot, it was winter, I was barefoot, without a coat, without a bag, without IDs… I lost everything! When everyone found me, and took me to the hospital I was kind of apathetic.

(Bianca)

These exemplary cases resonate with Minkowski's (2004) loss of vital contact with reality. The house keys lose their meaning as an opening to the home; cold and hunger are not felt by the numb body. The intentionality that unites the person to the world is suspended. The body wanders aimlessly (“I lost my shoes”), and consciousness remains imprisoned in a sphere, inaccessible to oneself and to others. It is the final metamorphosis into an existence where memory fades and intentional agency disappears.

### 4.6 The Invariant of Disentanglement: Without a Body–Consciousness–World Rupture, There Is No Lived Illness

The imaginative variation of the shared experiential descriptions allows us to glimpse an invariant traversing them all: severe mental illness only truly becomes a lived illness when a painful rupture in the body–consciousness–world intertwining takes hold.

Despite the phenomenological diversity of the illustrative episodes summoned—Roberto's *fully stretched heart*, João's gravitational bed, Mafalda's impossible smells, Maria's persecutory gazes and laughter, Mário's *planet madness*, and Bianca's blackout—the same structural fracture of embodied existence is evident across all of them. Regardless of the specific manifestation or singularity of the lived experience, the essence of this experience resides in the collapse of the transparency uniting body, consciousness, and world: the body ceases to be a silent ally, becoming an obstacle or threat, while the world ceases to offer familiarity, presenting itself as strange or inaccessible.

If we imagine, through free variation, the removal of this rupture—restoring the fluidity of the intentional arc and the *silence of the organs*—the phenomenon of the lived experience of illness (*illness*) disappears, leaving behind, perhaps, only the biological condition (*disease*).

It is this rupture, rather than a checklist of symptoms or diagnostic labels, that configures the ontological core of illness as a lived experience. To state that without a body–consciousness–world rupture there is no lived illness is to affirm that psychopathology is not merely a quantitative deviation from normality, but a disentanglement taking multiple forms of metamorphosis in one's way of inhabiting the world. The human being is forced to inhabit the rubble of a relationship that was once tacit.

## 5 Discussion

The unveiled illustrative episodes and their respective reflective analysis show that the lived experience of severe mental illness transcends the symptomatological taxonomy of diagnostic manuals, manifesting as a radical transformation in the fundamental structure of being‐in‐the‐world. The essential theme, “a disentanglement in multiple forms of body–consciousness–world metamorphosis,” dialogues phenomenologically with the experience of mental illness as a stalled existence (Telles and Moreira 2014), where the pre‐reflective unity of the person's experience suffers a violent rupture.

### 5.1 From the Slackening of the Intentional Arc to Disentanglement: An Ontological Amplification

The metamorphosis unveiled in the illustrative episode dialogues directly with the Merleau‐Pontyan perspective that illness presents itself as a slackening of the intentional arc (Merleau‐Ponty 2020). In health, this arc projects existence into a pre‐reflective fluidity that unifies body, consciousness, and world. The body effaces itself in its transparency to allow access to the world.

However, in the experience of severe mental illness, there occurs not merely a punctual slackening, but a structural metamorphosis that we term *disentanglement*. The eidetic findings unveil that this slackening appears as a radical fracture of the chiasmic tissue: the body ceases to be an ally to become a threat, consciousness loses its temporal continuity, and the world collapses into strangeness.

Roberto's *fully stretched heart*, for example, manifests not merely a dysfunctional body, but the simultaneous collapse of the embodied triad, namely the body that pulses against itself, the consciousness invaded by voices, and the world that withdraws into confusion. It also vividly illustrates what Toombs (1987) identifies as the loss of totality. The body ceases to be the *silence of the organs* and becomes a noisy opponent, an alienating presence that escapes the control of the will. The stretched heart presents itself as the ontological experience of a body that autonomizes itself as a threat, rupturing the body‐self unity and inaugurating a relationship of strangeness with oneself.

Thus, the notion of disentanglement presents itself as an ontological amplification of the Merleau‐Pontyan concept of slackening, seeking to describe and signify the lived experience of the person with severe mental illness.

### 5.2 The Oscillation Between Corporealization and Disembodiment

The way each of the lived experiences presented themselves confirms the pertinence of the poles described by Fuchs (2005) for understanding the variations of the metamorphosis:

1. **Corporealization in withdrawal:** João's case (“I couldn't get out of bed”) exemplifies hyper‐embodiment. The body becomes heavy, a leaden object that resists intentionality (Fuchs 2005). The degradation of everyday know‐how reflects what Carel (2016) defines as a loss of freedom: the horizon of possibilities closes, and the present transforms into an existential cell where the future is inaccessible. The bed, paradoxically comfortable and depressing, becomes the locus of a stagnant temporality.
2. **Disembodiment and the blackout:** In contrast, the experiences of Mário and Bianca, captured in rich expressions like “planet madness” and “blackout in the mind,” illustrate discarnation or disembodiment (Fuchs 2005; Spencer et al. 2025). Here, an approximation occurs with what Minkowski (2004) describes as the loss of vital contact with reality. The moving wave that unites the personality to the world (lifeworld) halts. Consciousness is lived as being enclosed in a sphere or on another planet, while the body wanders in an automatism devoid of ipseity. This separation is not a metaphor, but a fracture in operative intentionality: the house keys lose their meaning as an opening, and the cold is not felt, unveiling a profound existential anesthesia.

### 5.3 Corporeal Doubt and the Permeability of the Self

Mafalda's experience (“Very strange things”) brings to light the concept of corporeal doubt proposed by Carel (2016). Distinct from intellectual doubt, this is a radical somatic uncertainty: the tacit trust in the reliability of the senses crumbles. The permeability of boundaries (impossible smells, invisible touches) summons a dissolution of the protective barrier between the self and the world. The lived body ceases to be a secure anchor to become an invaded territory.

### 5.4 The Crisis of Intercorporeality

The metamorphosis of the relationship with the social world, lived by Maria (“Everyone is looking at me,” “laughing and speaking ill of me”), demonstrates the collapse of Merleau‐Ponty's intercorporeality. The musical memory of interaction (Fuchs 2017) falls out of tune. The reversibility of intentions inverts: the gaze of the Other, which should confirm existence, becomes objectifying and persecutory. Laughter at the movies ceases to be sharing to become judgment. This alteration in the social flesh describes isolation not as a choice, but as a necessary defense of a *frightened animal* facing an alterity that has become hostile. The unveiled failure of intercorporeality calls for the reconstruction of a therapeutic relationality that functions as a safe harbor against the hostility of the social world.

In sum, the partial findings indicate that severe mental illness is, in its essence, a lived experience of a crisis of familiarity. Disentanglement is also the painful process where the world ceases to be a home to become an inhospitable space.

The identification of the eidetic invariant corresponding to the rupture of the body–consciousness–world unity challenges traditional biomedical logic. While psychiatric nosology tends to classify pathology as a quantitative deviation from normality (excess or deficit of functions), the unveiling of the essential theme “a disentanglement in multiple forms of body–consciousness–world metamorphosis” shows that the lived experience represents a qualitative metamorphosis. The suffering resides not only in the severity of symptoms, but in the way these configure the ontological core of the illness: a collapse of transparency that forces the human being to inhabit the rubble of a relationship that was once tacit. This confirms that, phenomenologically, the lived experience of illness (*illness*) only emerges when the pre‐reflective flow ruptures, distinguishing itself radically from the mere biological condition (*disease*).

Although the focus of this article is the disentanglement in multiple forms of metamorphosis, it is important to note that this is not a static state, but part of a non‐linear itinerary that also includes attempts at re‐intertwining and reconciliation (Brijan et al. 2025; Fusar‐Poli et al. 2022).

### 5.5 Study Limitations

As an inquiry anchored in the phenomenology of practice, traditional qualitative rigor criteria such as transferability, factual validation by participants, or auditability via software tools do not constitute limitations here, since the method's purpose is not empirical generalization or technical standardization, but the unveiling of the meaning of the experience in its essential structure (Van Manen 2014, 2023).

However, this study presents ontological and methodological challenges inherent to phenomenology itself. The central limitation resides in the ineffable nature of the pre‐reflective experience of severe mental illness. The collapse of the body–consciousness–world intertwining unveils itself as a radical and pathic rupture, often experienced before or beyond language. Thus, the need to translate this strangeness and corporeal opacity into verbal language during the conversational interview constitutes a challenge; the word may fail to capture the totality and depth of the lived metamorphosis.

Additionally, since the interviews occurred at a time when participants were clinically stabilized, the description of the rupture experiences presupposes a temporal and reflective distance. Although the phenomenological interview seeks a return to concrete experience, the *a posteriori* account always demands continuous rigor from the researcher in *epoché* and experiential reduction, to prevent rationalizations from obscuring the originary way the phenomenon gave itself to consciousness.

Finally, it is recognized that unveiling the disentanglement captures only one facet of this phenomenon, focusing on the moment of rupture. Future phenomenological studies may build upon this work by exploring a broader itinerary of lived experience, namely the attempts at re‐intertwining and the processes of reconciliation with a metamorphosed existence across various care settings.

## 6 Conclusions

Drawing from this phenomenological‐hermeneutic study and the unveiling of the essential structure of disentanglement in severe mental illness, mental health and psychiatric nursing is challenged to embrace a practice of *reverence for life* (Praeger 2000), focused on the restoration of the dialogue between the flesh of the body and the flesh of the world. This safeguards the transition from a symptom surveillance model to a clinical practice of presence and ontological mediation, rendering the nursing intervention containing and reparative.

### 6.1 From Diagnosis to Ethical Welcoming

The first implication is ethical. Faced with the strangeness of the metamorphosis—the body that smells bad for no reason, the fear of others' laughter—the nurse is summoned to practice Levinas's (2014) unconditional hospitality. The Face of the person cared for, in their radical vulnerability, imposes a responsiveness that precedes any clinical categorization. Welcoming alterity demands the practice of *epoché*: suspending judgment about madness to validate the lived truth of the suffering.

### 6.2 Listening as a Fusion of Horizons

Methodologically, mental health and psychiatric nursing can reinforce phenomenological listening as a core clinical competency. Transcending data collection for the initial assessment, this enables the nursing intervention to be a contemplative lingering (Han 2023). The nurse must listen to the body's metaphors (*fully stretched heart*, *blackout*) as precise descriptions of a lived experience that manifests the disruption of the fundamental bonds uniting body–consciousness–world. This listening allows for a fusion of horizons (Sholokhova et al. 2022), where subjective experience is legitimized as essential knowledge for the care plan.

### 6.3 Nursing as Re‐Intertwining

In advanced practice, the mental health and psychiatric specialist nurse can foster and attend to the need to promote re‐intertwining. Their specific and differentiated competencies gain new depth in this light through interconnected interventions. This encompasses the modulation of atmospheres, where the creation of safe corners and the modulation of the therapeutic environment (García 2024) are vital to reduce the threatening permeability lived by patients like Mafalda, restoring a minimal sense of boundaries and corporeal safety. Concurrently, it involves bodywork, integrating interventions focusing on bodily awareness and the rehabilitation of motor intentionality that can help combat both corporealization (heaviness) and disembodiment, assisting the person cared for to inhabit their body once again (Fuchs and Röhricht 2017). Furthermore, these competencies demand the mobilization of care horizons that reinforce a sense of hope, summoning the possibilities of being‐in‐the‐world. In the transition from a stalled existence to a new configuration, nursing stimulates the process of embodied sense‐making, assisting the person cared for to reinterpret and re‐inhabit their world and their subjectivity in mental health (McCaffrey 2024).

The focus must shift toward the rehabilitation of motor and pathic intentionality, allowing the patient to recover, even if in a metamorphosed way, the capacity to inhabit their own body and the shared world. The nurse thus affirms themselves as the professional who stands alongside the structural fracture of existence, helping to weave, thread by thread, a re‐intertwining into new possibilities of being‐in‐the‐world.

Humanistic nursing (Paterson and Zderad 1988; Watson 2002) presents itself here as the caring science that accompanies the person at the threshold of pain and metamorphosis. If illness can be experienced as a violent disentanglement, nursing is the patient art of helping to gather and integrate anew.

If the essence of severe mental illness manifests as the disentanglement of the tissue uniting body, consciousness, and world, nursing affirms itself through its ethical vocation to repair this bond. This study calls for a repositioning of the specialist nurse as a co‐inhabitant of a fractured world. By sustaining the intentional arc through a pathic presence, the nurse validates the ontological vulnerability of the Other, presenting themselves as a mediator in the reconstruction of relationality.

In this perspective, caring is configured as the art of helping to gather and weave, thread by thread, a new re‐intertwining between the flesh of the body and the flesh of the world, allowing existence, even in metamorphosis, to find new possibilities of being‐in‐the‐world.

## Artificial Intelligence Disclosure

The AI language model Google Gemini was used strictly as a supportive tool for translation and stylistic refinement. Specifically, it provided initial suggestions for the conversion of previously segmented lists into discursive prose, a modification requested by the Editor‐in‐Chief during the first round of review to align the manuscript with the journal's stylistic requirements. The AI outputs were treated solely as stylistic inspiration; the authors critically evaluated, altered, deepened, and determined exactly which suggestions to incorporate. To preserve methodological rigor and authorship, the construction of the entire article, the core phenomenological analysis, the *epoché*, the eidetic reduction, the interpretation of lived experiences, and the vocative writing were conducted entirely and manually by the authors. The authors take full responsibility for the integrity and originality of the final content.

## Ethics Statement

The study was approved by the institutional Ethics Committee (Process 3624/2021/CE). Free and informed consent was obtained from all participants.

## Conflicts of Interest

The authors declare no conflicts of interest.

## Data Availability

The data that support the findings of this study are available from the corresponding author upon reasonable request. The data are not publicly available due to privacy or ethical restrictions, as they contain information that could compromise the privacy of research participants.
